# Defining ‘Unhealthy’: A Systematic Analysis of Alignment between the Australian Dietary Guidelines and the Health Star Rating System

**DOI:** 10.3390/nu10040501

**Published:** 2018-04-18

**Authors:** Alexandra Jones, Karin Rådholm, Bruce Neal

**Affiliations:** 1 The George Institute for Global Health, Sydney 2042, Australia; kradholm@georgeinstitute.org.au (K.R.); bneal@georgeinstitute.org.au (B.N.); 2 Charles Perkins Centre, University of Sydney, Sydney 2006, Australia; 3 Division of Community Medicine, Primary Care, Department of Medicine and Health Sciences, Faculty of Health Sciences, Department of Local Care West, County Council of Östergötland, Linköping University, 581 83 Linköping, Sweden; 4 Department of Epidemiology and Biostatistics, School of Public Health, Faculty of Medicine, Imperial College London, London SW7 2AZ, UK

**Keywords:** nutrient profiling, front-of-pack labelling, dietary guidelines, nutrition policy, health star rating

## Abstract

The Australian Dietary Guidelines (ADGs) and Health Star Rating (HSR) front-of-pack labelling system are two national interventions to promote healthier diets. Our aim was to assess the degree of alignment between the two policies. Methods: Nutrition information was extracted for 65,660 packaged foods available in The George Institute’s Australian FoodSwitch database. Products were classified ‘core’ or ‘discretionary’ based on the ADGs, and a HSR generated irrespective of whether currently displayed on pack. Apparent outliers were identified as those products classified ‘core’ that received HSR ≤ 2.0; and those classified ‘discretionary’ that received HSR ≥ 3.5. Nutrient cut-offs were applied to determine whether apparent outliers were ‘high in’ salt, total sugar or saturated fat, and outlier status thereby attributed to a failure of the ADGs or HSR algorithm. Results: 47,116 products (23,460 core; 23,656 discretionary) were included. Median (Q1, Q3) HSRs were 4.0 (3.0 to 4.5) for core and 2.0 (1.0 to 3.0) for discretionary products. Overall alignment was good: 86.6% of products received a HSR aligned with their ADG classification. Among 6324 products identified as apparent outliers, 5246 (83.0%) were ultimately determined to be ADG failures, largely caused by challenges in defining foods as ‘core’ or ‘discretionary’. In total, 1078 (17.0%) were determined to be true failures of the HSR algorithm. Conclusion: The scope of genuine misalignment between the ADGs and HSR algorithm is very small. We provide evidence-informed recommendations for strengthening both policies to more effectively guide Australians towards healthier choices.

## 1. Introduction

Unhealthy diets—high in salt, harmful fats, added sugar and energy—are a leading cause of death and disability in Australia [1]. Australia has some of the highest obesity rates in the world: nearly two-thirds of Australian adults and one in four children are now overweight or obese. Unprecedented availability and aggressive marketing of processed and pre-packaged foods and beverages are a key driver of obesity and diet-related conditions including high blood pressure, heart disease, stroke, type 2 diabetes, some forms of cancer, dementia and dental caries [2]. Obesity alone is estimated to cost Australia more than $8.6 billion annually [3].

The World Health Organization recommends a comprehensive suite of population health approaches to promote healthier diets. These include laws and regulations, tax and price interventions, community-based measures in facilities such as schools and hospitals, and public education through social marketing campaigns [4]. Despite the increasing impact of poor diet on Australia’s health, few of these preventive strategies have been taken up at a federal level.

Two policy areas where Australia has been benchmarked as performing well against international best-practice are in adoption of food-based dietary guidelines and front-of-pack nutrition labels [5]. The current Australian Dietary Guidelines (ADGs) were introduced in 2013 to promote health and wellbeing while reducing the risk of chronic disease [6]. In 2014, Australia adopted the Health Star Rating System (HSR), an interpretive nutrition labelling scheme that rates foods from 0.5 to 5.0 stars on the front-of-pack [7]. An example of the HSR graphic and the Australian Guide to Healthy Eating, which provides a practical visual representation the food groups recommended by the ADGs and their proportions, are included at Appendix A.

While inherently related in their intent to guide Australians towards healthier choices, the two measures differ in aspects of their purpose and design (Table 1). For example, the ADGs provide information on dietary patterns, amounts and food groups that support health through detailed guidance documents that are for use by health professionals, policy makers, educators, food manufacturers and researchers [6]. By contrast, HSR uses an algorithm to quantify selected aspects of individual foods, generating a summary score displayed in a simple symbol intended to both target consumers at the point-of-sale, and offer incentives for manufacturers to improve recipes to receive a higher rating [8,9]. The relative strengths of each measure suggest potential opportunities for the ADGs and HSR to operate synergistically for maximum public health impact. At the same time, tension between the two approaches is apparent in academic and media critique of HSR, particularly where high profile products have been highlighted for displaying labels allegedly inconsistent with ADG recommendations [10,11].

With a five year review of the HSR system currently underway, our objective was to assess one of the review’s key elements, namely the degree of alignment between the two policies and specifically in their mechanism for defining food as healthy or unhealthy. Policy coherence is important not only because of the need to provide consistent dietary messaging to Australians, but also because inappropriate HSR scores or ADG recommendations threaten the credibility and sustainability of both policies. By identifying areas and causes of misalignment, our aim was to make evidence-informed recommendations for refining both policies, thereby strengthening Australia’s efforts to promote healthier diets.

## 2. Methods

This was a cross-sectional examination of packaged foods and beverages (hereafter referred to as foods) available in Australia.

### 2.1. Data Source

We analysed items included in The George Institute for Global Health’s Australian FoodSwitch Database [12]. The database contains nutrition label information from packaged foods systematically collected by The George Institute through large-scale annual surveys, as well as provided directly by manufacturers and consumers on a rolling basis. In total, this data represents more than 90% of products available in the Australian market. For this study, we used information extracted directly from the back-of-pack nutrition information panel. Energy (kJ/100 g), protein (g/100 g), saturated fat (g/100 g), total sugar (g/100 g), and sodium (mg/100 g) are mandatory on the Australian nutrient declaration but details on fruit, vegetable, nut and legume (FVNL) (%), concentrated FVNL (%), and fibre (g/100 g) are optional. Where such details were absent, appropriate levels were estimated using information drawn from the back-of-pack ingredients list, generic food composition databases, or by analogy with similar products using methods described previously [12]. The estimation process provides a proxy value for each nutritional indicator at the finest category level for more than 1000 individual food subcategories. Proxy values are then substituted for each product in that category for which data are missing.

### 2.2. Product Classification

Classification of products was based on the system developed by the Global Food Monitoring Group and incorporated into FoodSwitch [13]. This hierarchical system is designed to monitor the nutrient composition of processed foods around the world. It classifies foods into major categories (e.g., bread and bakery products), categories (e.g., bread), and subcategories (e.g., pita bread). Our analysis included only packaged food items. We excluded infant foods and formula, vitamins and supplements, formulated supplementary sports foods, foods for special medical purposes and alcoholic beverages because these foods have been specifically deemed outside the scope of HSR [14]. This left 15 major categories for analysis. Within these, we also excluded subcategories of plain tea and coffee, herbs and spices, baking powders, yeasts and gelatines, as these foods do not contribute significantly to nutrient intake, are not required to display a nutrition information panel [15], and are also therefore not required to display a HSR.

### 2.3. Calculation of the Health Star Rating

The HSR was calculated in alignment with the methods described in the ‘Guide for industry to the Health Star Rating Calculator’ for all products sold, regardless of whether a HSR was reported on the pack [16]. In short, foods were categorised into one of six HSR categories (i.e., non-dairy beverages; dairy beverages; oils and spreads; cheese and processed cheese; all other dairy foods; all other non-dairy foods). Baseline points were calculated based on the energy, saturated fat, total sugar, and sodium content per 100 g. Modifying points for FVNL%, concentrated FVNL%, protein, and fibre were calculated, where applicable. A HSR ‘score’ was calculated by subtracting the modifying points from baseline points. This score is then converted to a HSR based upon a defined scoring matrix for each of the six categories [16]. The HSR ranges from 0.5 to 5.0 stars in ten half-star increments. A higher HSR reflects a healthier product.

### 2.4. Classification of Foods under the Australian Dietary Guidelines

We classified foods as ‘core’ or ‘discretionary’ according to ADG guidance. In short, core foods were defined as those from the ADGs Five Food Groups: grain (cereal) foods, mostly wholegrain and/or high cereal fibre varieties; vegetables and legumes/beans; fruit; milk, yoghurt, cheese and/or alternatives, mostly reduced fat; and lean meats and poultry, fish, eggs, tofu, nuts and seeds and legumes/beans. Together, these core foods form the basis of a healthy diet. Discretionary foods are those not necessary to provide nutrients the body needs, and are defined for ADG purposes as those ‘high in’ saturated fats, added sugars, and/or salt or alcohol [6]. As the ADG documents themselves provide only limited examples of discretionary choices and no objective measure of ‘high in’, we relied upon the Australian Bureau of Statistics (ABS) ‘Discretionary Food List’ [17] as the best-available reference for classifying each product for the purposes of this analysis. The main principle used by the ABS to classify foods as discretionary is that they were specified or inferred in the ADGs and supporting documents as discretionary [17]. ABS classifications determined at a detailed food category level were matched to FoodSwitch categories to classify each category as ‘core’ or ‘discretionary’.

In some categories, such as those involving mixed foods, the ABS applies additional nutrient criteria to define core and discretionary. The nutrient cut-offs specified are those used in the modelling that supported the original ADG development [18]. Where provided, e.g., pizza with saturated fat content ≤5 g/100 g is ‘core’ while pizza with saturated fat content >5 g/100 g is ‘discretionary’, these were also applied.

### 2.5. Statistical Analysis

Cross-tabulations of ADG status and HSR were prepared for the twenty cells comprising core, discretionary and the ten possible HSR values (0.5, 1.0, 1.5, 2.0, 2.5, 3.0, 3.5, 4.0, 4.5 or 5.0). In the absence of endorsed HSR cut-offs for healthy or unhealthy, we identified products as ‘apparent outliers’ when the product was categorised ‘core’ by the ADGs but received a HSR ≤ 2.0, suggesting an unhealthy nutritional profile, or the product was categorised as ‘discretionary’ by the ADG but received a HSR ≥ 3.5, suggesting a healthy nutritional profile. The number and proportion of products deemed apparent outliers was determined overall, for each of the 15 major food categories included and by category and sub-category where helpful.

To further understand the reasons for outlier status of products, and in particular the potential impact of the undefined ‘high in’ terminology used by the ADGs, we applied additional nutrient cut-off criteria. In the absence of any existing international standard or guidance, we drew from the United Kingdom (UK) multiple traffic-light nutrient profile model, which was validated during development in the UK context, and has been subsequently used to model dietary outcomes elsewhere [19,20,21]. Specifically, the cut points used to apply red traffic lights for salt, total sugar and saturated fat were used to provide a quantitative measure of the ADGs ‘high in’ terminology. These were applied to apparent outliers, and products were removed or retained on the basis of qualifying for none, one or multiple red traffic lights:

- Apparent outliers that scored a low HSR but were assigned ‘core’ status by the ADGs were not considered ‘genuine outliers’ if they were sufficiently ‘high in’ salt, saturated fat and/or sugar to warrant at least one red traffic light.
- Apparent outliers that scored a high HSR but were assigned ‘discretionary’ status by the ADGs were not considered ‘genuine outliers’ if nutrient values for salt, saturated fat and/or sugar were not sufficiently high to warrant at least one red traffic light.

Apparent outliers that were deemed not to be genuine outliers after application of these ‘high in’ cut points were deemed ‘ADG failures’. All others were deemed ‘HSR failures’. Reasons for failures and potential solutions were systematically recorded (Appendix B).

## 3. Results

In total, 65,660 packaged products available in Australian supermarkets between 1 January 2013 and 30 June 2017 were identified in the FoodSwitch database. Of these, 11,431 had insufficient product information to enable categorization and generate a HSR, and a further 7113 were in categories excluded from HSR.

This left 47,116 products for analysis. Of these, only 3524 (7.5%) were displaying HSR on pack at the date of data extraction (30 June 2017).

### 3.1. HSR Distribution by Core and Discretionary

**Figure 1:**
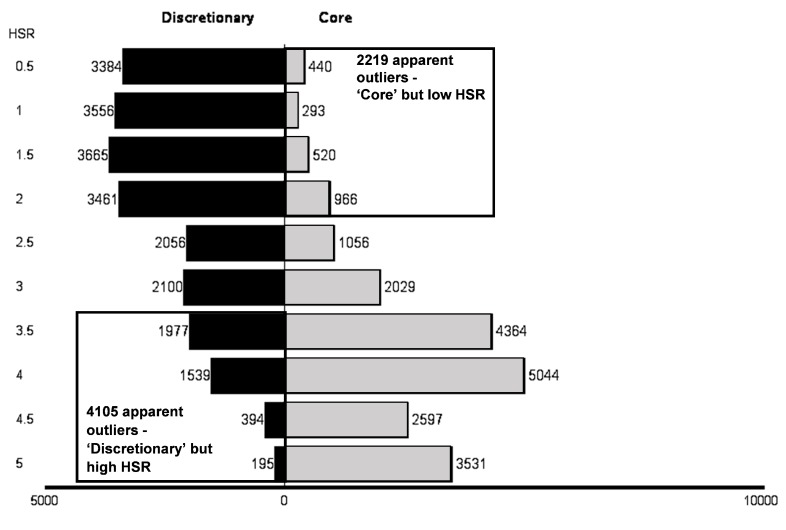
Distribution of HSR by core and discretionary with apparent outliers.

There were 23,460 (49.8%) core products and 23,656 (50.2%) discretionary products. Distribution of HSR by core and discretionary is shown in Figure 1.

In total, the median (IQR) of calculated HSR scores was 3 (1.5 to 4). Core products had a median (IQR) calculated HSR score of 4.0 (3.0 to 4.5) and discretionary products had a median (IQR) calculated HSR score of 2.0 (1.0 to 3.0).

Of products displaying HSR on pack, the overall median (IQR) value was 4.0 (3.0 to 4.5); out of these, 2131 (60.5%) were core products and 1393 (39.5%) were discretionary.

### 3.2. Apparent Outliers

There were 6324 (13.4%) apparent outliers. In total, 2219 were apparent core outliers, representing 9.5% of all core products and 4.7% of the total sample. In total, 4105 apparent discretionary outliers were identified, representing 17.4% of all discretionary products and 8.7% of the total sample (Figure 1 and Table 2).

**Figure 2:**
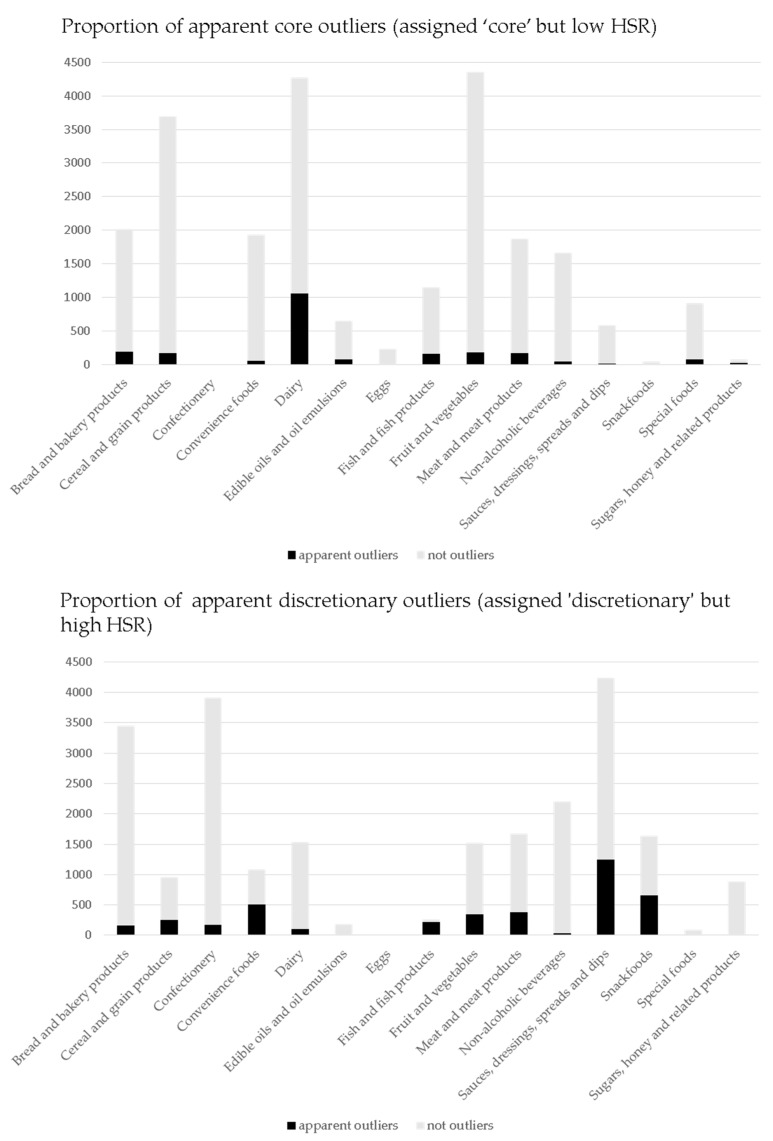
Apparent core and discretionary outliers—numbers of products and numbers of outliers by major food category.

As seen in Figure 2, the distribution of products and number of apparent outliers varied greatly across the 15 major food categories and by core and discretionary classification. The major categories with the largest proportion of apparent outliers were sauces, dressings, spreads and dips (19.9%); dairy (18.4%); and snack foods (10.3%).

### 3.3. Application of Traffic Light Cut-Offs

Application of cut-points to identify foods ‘high in’ salt, total sugar and saturated fat greatly reduced the number of outliers (Table 2).

**Figure 3:**
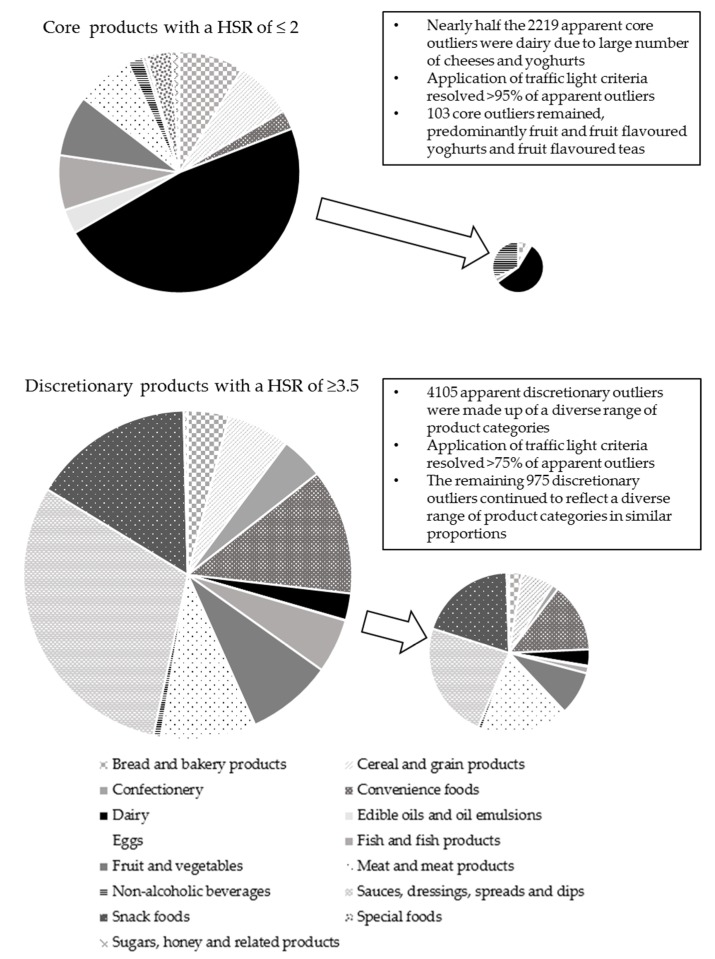
Apparent and genuine outliers by major food category (areas of circles are proportional to numbers of products).

Among the apparent core outliers, 2116/2219 (95.4%) had at least one red traffic light, signifying high levels of salt (1159), saturated fat (1136), and/or sugar (538). These foods were deemed ADG failures on the basis that high levels of these negative nutrients form the basis of the ADG definition of discretionary foods. This left 103 (4.6%) core food outliers that received a low HSR despite not being ‘high in’ any negative nutrients. These results were genuinely misaligned with the ADGs and deemed HSR failures. Figure 3 and Table 3 detail these results by major food category. The three categories with HSR failures were fruit and flavoured yoghurts; and flavoured teas. The yoghurts had amber lights for saturated fat and sugar, and the flavoured teas had amber lights for sugar. Both categories likely had a mix of naturally occurring and added sugar.

In the discretionary outlier group, 3130/4105 (76.2%) of apparent outliers had no red traffic lights, signifying they were not ‘high in’ salt, sugar or saturated fat and were therefore deemed ADG failures. This left 975 (23.8%) apparent discretionary outliers receiving a high HSR despite being ‘high in’ salt (510), sugar (296) and/or saturated fat (235). These foods were deemed HSR failures. Figure 3 and Table 4 outline these findings by major food category. The largest number of HSR failures occurred in sauces, dressings, spreads and dips; savoury snacks; meat and meat products; and, convenience foods. Products in these categories predominantly had red traffic lights for salt and to a lesser degree, saturated fat despite receiving HSR ≥ 3.5.

Taking core and discretionary together, 5246 outliers (83%) were attributable to ADG failure. This left 1078 outliers (17%) attributable to a failure of the algorithm.

A detailed list of core and discretionary outliers is included at Appendix B.

## 4. Discussion

In contrast to intense media attention on occasional anomalies, this large quantitative analysis suggests that the scope of genuine misalignment between the ADGs and the HSR algorithm across the Australian food supply is very small.

The degree of policy coherence demonstrated by our results is encouraging, though perhaps not surprising given the well-recognised relationship between nutrients, foods and dietary patterns [22]. Our results are consistent with existing research demonstrating that the HSR algorithm is aligned well overall with the ADGs [23,24,25], and that the median HSR of core foods is significantly higher than that of discretionary foods [10,23,26].

While these results are promising, it is reasonable to seek better alignment between the HSR and the ADGs if this increases their public health impact. The results of this work suggest directions for improvement.

Specific recommendations for improving alignment are set out in Table 5.

While there is considerable current focus on review of the HSR algorithm, these results highlight a parallel urgent need for review of the ADG text and corresponding ABS Table (See Appendix B).

Our findings can be differentiated from a recent analysis of foods carrying HSR labels on pack during voluntary implementation [19]. That analysis of 1269 new products found that 57% were core items and 43% discretionary. The authors concluded that HSR labelling was undermining ADG recommendations by facilitating the marketing of discretionary foods because more than half of those defined as discretionary displayed a HSR ≥ 2.5. Our work suggests caution in this interpretation given the small sample size of the prior study, identified failings of the ADGs in regard to the classification of some foods, and the highly selective subset of products studied. The conclusions may also reflect the simplistic approach ultimately taken by the ADGs, in seeking to dichotomise foods as core or discretionary, when healthiness of products is almost certainly distributed along a continuum.

The present analysis benefits from the large number of foods included, their robust representation of the broader Australian food supply and the comprehensive and systematic approach taken to the evaluation and presentation of the data. Our decision to use the UK traffic light criteria as a quantitative measure of ‘high in’ was an objective approach to quantifying the textual guidance provided by the ADGs. The international food standards agency, The Codex Alimentarius Committee, recently agreed to commence work to develop ‘high in’ criteria for salt, sugar and saturated fat given increasing international interest in this area. This process is likely to take several years [27].

Some limitations also need to be mentioned. FVNL content and fibre are not currently mandatory on back-of-pack nutrition information panels in Australia and missing values were therefore estimated from ingredients lists, food composition databases, and other sources. This analysis does not capture related concerns raised by stakeholders to the Government’s five year review [28] regarding the high HSRs received by fruit juices, breakfast cereals with a sugar content ≤30 g/100 g, and breakfast beverages. These issues likely represent HSR failures but are not identified as ‘outliers’ in this analysis because the ADG text and/or ABS Table also identify these products (arguably incorrectly) as core.

Our ability to measure alignment was limited by the components of foods considered by the HSR algorithm. For example, HSR currently relies on total sugar but the ADGs recommend to limit added sugars specifically. Areas where we believe this distinction may have impacted outlier status include flavoured yoghurts and milks, breakfast cereals, muesli bars, chutneys and table sauces as noted in detail in Appendix B. Previous work has indicated that incorporating added sugar into the HSR algorithm would improve its ability to discriminate between core and discretionary [26,29]. This would be best facilitated by updating mandatory nutrient information panel (NIP) requirements to include transparent information on added sugars. Alternatively, added sugar values could be systematically estimated using available information from current NIPs in combination with the ingredients list, using published methods [30]. A similar approach is currently provided to companies claiming points for fruit, vegetable, nut and legume content that is also not required in the NIP.

Our analysis was also limited in scope to packaged products only. While half our sample were classified core (suggesting that not all packaged foods are unhealthy), some foods recommended by the ADGs (i.e., whole fresh fruit and vegetables) are generally sold without packaging. Current consideration of whether to extend HSR to these products (e.g., through shelf talkers) could further enhance alignment between the HSR and ADGs [31].

## 5. Conclusions

This work illustrates the complexity of defining foods as ‘healthy’ or ‘unhealthy’ across the huge range of packaged products available in the current Australian food supply.

Like other front-of-pack labelling systems that rely on an underlying profiling model, the HSR algorithm is intended as a tool to quantify selected aspects of individual foods rather than to be a complete source of dietary advice. Nevertheless, our results are consistent with WHO recognition that such tools are a helpful method to use in conjunction with interventions aimed at improving the overall nutritional quality of diets [32].

Rather than undue focus on perfect alignment or determination of the superiority of the HSR or ADGs, a more nuanced understanding of the relative contribution (and inherent limitations) of each suggests areas where the design and implementation of both policies could be strengthened to guide Australian consumers towards healthier choices.

## Figures and Tables

**Table 1:** Key features of Australian Dietary Guidelines and the Health Star Rating System.

	Objective	Mechanism	Target Audience	Classification of Foods	Developed by	Governed by
**Australian Dietary Guidelines**	Provide information on food groups, amounts and dietary patterns that support health.	Guideline Documents, Summary and Educator Guide. Australian Guide to Healthy Eating graphic	Health professionals, policy makers, educators, food manufacturers, food retailers and researchers.	Classification of foods into Five Food Groups that form the basis of a healthy diet, and ‘discretionary’ foods defined by the presence of saturated fat, added sugars, salt and/or alcohol, whose intake is to be limited.	National Health and Medical Research Council (NHMRC) via standardised guideline process. Working Committee incl. public health and industry representation.	NHMRC NHMRC considers whether to update after 5 years. Maximum interval prior to update is 10 years.
**Health Star Rating**	Simplify nutrition information available on back-of-pack to differentiate between individual foods more likely to be part of a healthy diet from those that are less healthy.	Front-of-pack label to be applied voluntarily by food retailers and manufacturers using relevant policy documents.	Consumers at point of purchase. Food retailers and manufacturers.	A nutrient profile model is used to score individual products from 0.5 to 5.0 stars. The algorithm considers energy, negative nutrients the ADGs recommend eating less of (saturated fat, sugars and sodium), and foods the ADGs recommend eating more of (fruits, vegetables, nuts and legumes) as well as in some instances, allowing points for protein and dietary fibre content.	Australian Fed., State and territory governments in partnership with food industry, consumer and public health groups.	Health Star Rating Advisory Committee. Representation from Australian Federal, State and Territory governments as well as food industry, consumer and public health groups. 2 year monitoring report and 5 year review process set down at adoption.

**Table 2:** Cross tabulation of apparent outliers, and attribution of reason for outlier status (i.e., ADG failure or HSR failure) after application of traffic light cut-offs.

	Apparent Outliers	ADG Failure	HSR Failure
HSR ≤ 2.0	2219	2116	103
HSR ≥ 3.5	4105	3130	795

**Table 3:** Core products with HSR ≤ 2.0.

Major Food Category	Number of Apparent Outliers	Number of Products with Any Red TLL	Outliers Remaining	Illustrative Examples of Outliers Remaining	Characteristics of Remaining Outliers
**1 Bread and bakery products**	193	187	6	Pancake mixes, tortillas	All have amber traffic lights for saturated fat and salt, and some also for sugar
**2 Cereal and grain products**	174	172	2	Rice puff cereal, noodles	Breakfast cereal has amber lights for salt and sugar Noodles have amber lights for saturated fat and salt
**3 Confectionery**	0	-	-		-
**4 Convenience foods**	57	56	1	Antipasto product	Has amber traffic lights for salt, saturated fat and sugar
**5 Dairy**	1056	997	58	Fruit and flavoured yoghurts, natural yoghurt	All are in yoghurt category. More than 90% are yoghurts with fruit or flavourings that have amber traffic lights for saturated fat and sugar Two products are natural yoghurts with amber traffic lights for saturated fat and salt
**6 Edible oils and oil emulsions**	73	73	0		-
**7 Eggs**	0	-	-		-
**8 Fish and fish products**	163	160	3	Salmon pate, garlic prawns	All have amber lights for salt and at least one other of sugar and saturated fat
**9 Fruit and vegetables**	181	181	0		-
**10 Meat and meat products**	168	168	0		-
**11 Non-alcoholic beverages**	46	13	33	Fruit flavoured teas and iced teas, matcha	Some teas, unlike most, carried a nutrient information panel and therefore had a HSR generated despite being low in nutrients overall Iced teas with added sugar are discretionary but these teas contained fruit and in the absence of added sugar labelling it was not possible to definitively categorise these drinks as core or discretionary Growth in popularity of new beverages categories (e.g., matcha, chai) suggest more classification guidance needed
**12 Sauces, dressings, spreads and dips**	11	11	0		-
**13 Snack foods**	0	-	-		-
**14 Special foods**	75	75	0		-
**15 Sugars, honey and related products**	23	23	0		-
**All**	**2219**	**2116**	**103**		

TLL = Traffic Light Label, referring to the threshold set for a red traffic light under the UK nutrient profiling model.

**Table 4:** Discretionary products with HSR ≥ 3.5.

Major Food Category	Number of Apparent Outliers	Number of Products No Red TLL	Outliers Remaining	Illustrative Examples of Outliers Remaining	Characteristics of Remaining Outliers
**1 Bread and bakery products**	166	141	25	Sweet biscuits, savoury breads and pastries	Products have red TLL for sugar (sweet breads and biscuits), salt (savoury breads and biscuits) or saturated fat (puff pastries, quiche)
**2 Cereal and grain products**	254	190	64	Breakfast cereals, cereal and nut-based bars	Most have red TLL for sugar and a few salt or saturated fat
**3 Confectionery**	173	161	12	Jellies, cocoa powder, chocolate strawberries	Products have red TLL for sugar (jellies) or saturated fat (chocolate based items)
**4 Convenience foods**	508	372	136	Ready meals, meal kits	Most products have red TLL for saturated fat and/or salt A smaller number have red TLL for sugar
**5 Dairy**	108	76	32	Rice puddings	All products have red TLL for sugar
**6 Edible oils and oil emulsions**	2	0	2	Almond oil, lemon butter	All products have red TLL for saturated fat or sugar.
**7 Eggs**	0	-	-		-
**8 Fish and fish products**	219	206	13	Salt and pepper products, fish cakes	All products have red TLL for salt.
**9 Fruit and vegetables**	347	261	86	Fruit bars and bites, pickled vegetables	Fruit products have red TLL for sugar (fruit bars, bites) and sometimes saturated fat (fruit bites with coconut) Vegetable products have red TLL for salt (pickled vegetables, olives)
**10 Meat and meat products**	384	213	171	Sliced meats, frozen and chilled meats	Most products have red TLL for salt or saturated fat and a few for both
**11 Non-alcoholic beverages**	30	22	8	Milk flavourings, beverages mixes	Products have red TLL for sugar. Are able to take advantage of ‘as prepared’ rules
**12 Sauces, dressings, spreads and dips**	1245	1015	230	Salty dips, relishes and chutneys	Most products have red TLL for salt, some for sugar and a few for saturated fat
**13 Snack foods**	652	461	191	Potato chips, vegetable and legume-based snacks, corn chips	Most products have red TLL for salt, some for saturated fat, a few for sugar and a few for several
**14 Special foods**	2	2	-		-
**15 Sugars, honey and related products**	15	10	5	Syrups	Products all have red TLL for sugar
**All**	**4105**	**3130**	**975**		

TLL = Traffic Light Label, referring to the threshold set for a red traffic light under the UK nutrient profiling model.

**Table 5:** Priority recommendations for reviewing the HSR algorithm and ADG definitions to improve alignment.

Review the weighting given to salt in HSR algorithm, given the large number of sauces, dips, savoury snacks, sliced meats and convenience foods that receive a high HSR despite being high in salt. This would be supported by the 2017 update of Australia’s Nutrient Reference Value for sodium.
Review the eligibility of fried or pickled vegetables and dried fruits for FVNL points given their receipt of high HSR scores despite being high in negative nutrients. This would be supported by references in ADG text (but not the ABS Table) that such products should be only consumed occasionally and in small amounts.
Review the weighting given to sugar, and/or incorporate added sugars given the large number of outliers in categories likely to contain a mix of naturally occurring and added sugars. These appeared at both ends, i.e., core yoghurts with fruit or flavours, fruit flavoured teas, as well as discretionary chutneys, breakfast cereals, muesli and fruit bars, dairy desserts and table sauces.
Review the ADG definition of discretionary, including additional guidance on ‘high in’ criteria for saturated fat, added sugars and salt to elucidate, for example, at what point a flavoured yoghurt can more properly be considered a dairy dessert.
Review a wide range of ABS table classifications (see Appendix B) including: ‘core’ status of breakfast cereals with sugar up to 30 g/100 g, cheese regardless of salt and saturated fat content, yoghurt and flavoured milks regardless of sugar or saturated fat content, most meat and fish regardless of salt content; ‘discretionary’ status of vegetable and legume based dips, ‘potato products’ and crumbed fish intended for home baking; and, appropriate treatment of growing product categories such as breakfast beverages and coconut products.
Once the algorithm is reviewed, make HSR mandatory to enable consumers to receive the full benefit of the system’s performance across the food supply.

## Appendix A. Australian Health Star Rating graphic and Australian Guide to Healthy Eating

(a) Health Star Rating system graphic

Examples of the HSR graphic to be applied to the front of packaged foods. The label can be applied in multiple acceptable configurations, with and without the addition of energy content and select nutrient information (in addition to the nutrient information panel mandated on the back of pack).

Source: Health Star Rating System Style Guide [14], available at: http://healthstarrating.gov.au/internet/healthstarrating/publishing.nsf/Content/651EEFA223A6A659CA257DA500196046/$File/HSR%20Style%20Guide-v5.pdf (accessed 8 April 2018).

(b) The Australian Guide to Health Eating (AGHE)

The AGHE is a food selection guide and the primary education and promotional tool in the Australian Government’s Eat for Health Program [33]. It converts scientific knowledge of food composition and nutritional requirements for optimal health and wellbeing into a practical guide representing the proportion of ADGs Five Food Groups recommended for consumption each day.

For the purposes of this paper, Five Food Group foods, appearing within the circular ‘plate’ are referred to as ‘core’ foods. Foods in the bottom right corner stated to be for consumption ‘only sometimes and in small amounts’ are illustrative examples of ‘discretionary’ foods.

Source: Australian Government Eat for Health Website, available at https://www.eatforhealth.gov.au/guidelines/australian-guide-healthy-eating (accessed 8 April 2018).

## Appendix B. Core and Discretionary Outliers by Major Food Group, Category, Traffic Light, ADG/HSR Failure and Policy Recommendation

(a) Core foods receiving HSR ≤ 2.0

**Major Food Categories *(Extracts of Relevant ADG Text *)***	***n***	**Saturated fat Traffic Light Red**	**Sodium Traffic Light Red**	**Total Sugar Traffic Light Red**	***n* ADG Failure**	***n* HSR Failure**	**Policy Recommendation (Where *n* ≥ 10)**
**Bread and Bakery**							
*Guideline 2: Go for wholegrains. Wholemeal or wholegrain varieties are preferable because they provide more dietary fibre, vitamins and minerals than refined grain (cereal) foods...* *Grain (cereal) foods which have high amounts of added saturated fats, added sugars, and/or salt such as most cakes, muffins, pies, pastries and biscuits are not included in this group but are classified ‘discretionary’ choices.*							
Biscuits							
Savoury biscuits	76	49	65	8	74	2	Review ABS table, including kJ cut-off for biscuits
Plain dry biscuits	49	4	22	0	48	1	Review ABS table, including kJ cut-off for biscuits
Bread							
Flat bread	29	6	24	0	29	1	None. Less healthy options of core foods
Other bread	20	11	0	18	20	0	None. Less healthy options of core foods
Cakes, muffins and pastries							
Pancake mix	17	1	12	6	14	3	Review ABS table
Crepe mix	4	4	1	4	4	0	
**Cereal and grain products**							
*Guideline 2: Go for wholegrains. Wholemeal or wholegrain varieties are preferable because they provide more dietary fibre, vitamins and minerals than refined grain (cereal) foods...* *Grain (cereal) foods which have high amounts of added saturated fats, added sugars, and/or salt such as most cakes, muffins, pies, pastries and biscuits are not included in this group but are classified ‘discretionary’ choices.*							
Noodles							
Flavoured noodles	88	79	83	1	88	0	Review ABS table
Plain noodles	6	2	3	0	5	1	
Pastas							
Packet pastas	28	0	28	0	28	0	Review ABS table
Fresh filled pasta	10	10	4	0	10	0	
Plain dry pasta	3	0	3	0	3	0	
Other grains and cereals (e.g., breadcrumbs)	11	1	11	0	11	0	Review ABS table
Breakfast cereals							
Muesli	6	6	0	1	6	0	
Granola	4	4	0	2	4	0	
Flakes	3	0	3	0	3	0	
Sweet cereals	3	1	0	3	3	0	
Others	2	0	0	1	1	1	
Rice							
Packet flavoured rice	4	0	4	0	1	0	
Dry rice	1	0	1	0	1	0	
Cous cous	5	0	5	0	5	0	
**Confectionery**	-	-	-	-	-	-	
**Convenience foods**							
*Guideline 2: Eat a wide variety of nutritious foods from the five food groups…* *Guideline 3: Limit intake of foods containing saturated fats, added salt, added sugars and alcohol.*							
Pre-prepared salads and snacks							
Antipasto	14	4	12	1	13	1	Review ABS table
Others (salad, sushi, sandwich)	8	3	8	1	8	0	
Pizza	20	0	18	4	18	0	Review ABS table. Consider salt cut-off
Other frozen foods not specified	9	9	3	0	9	0	
Soups	3	0	3	0	3	0	
**Dairy**							
*Guideline 2: Include milk, yoghurt and cheese and/or alternatives—mostly reduced fat* *Full fat cheeses should be limited to 2–3 serves per week, and varieties which are lower in salt are preferable*							
Cheese							
Hard and semi-hard cheeses	283	283	270	0	283	0	Review ABS table, core status given salt and sat fat
Soft cheeses	177	177	73	0	177	0	Review ABS table, core status given salt and sat fat
Processed cheeses	30	30	30	0	30	0	Review ABS table, core status given salt and sat fat
Sheep/goat cheese	13	13	12	0	13	0	Review ABS table, core status given salt and sat fat
Soy cheese	9	9	9	0	9	0	Review ABS table, core status given salt and sat fat
Yoghurt							
Yoghurt with fruit	204	82	0	145	163	41	Review ABS table, core status given sat fat and sugar HSR failures suggest review sugar, added sugars
Flavoured yoghurt	105	64	0	75	95	10	Review ABS table, core status given sat fat and sugar HSR failures suggest review sugar, added sugars
Natural yoghurts	57	53	0	6	55	2	Review ABS table, core status given sat fat and sugar
Non-dairy yoghurts (coconut)	23	23	0	0	23	0	Review ABS table (Coconut products)
Yoghurt mixes	24	6	0	22	22	2	HSR impacted by ‘as prepared’
Yoghurts with muesli or non-fruit additions	19	11	0	18	19	0	Review ABS table, core status given sat fat and sugar Review sugar, added sugars in HSR algorithm
Drinking yoghurt	1	0	0	0	0	1	
Milks							
Coconut milks and creams	79	79	0	0	79	0	Review ABS table (Coconut products)
Dairy milks	19	15	0	15	19	0	Review ABS table, core status given sat fat and sugar Review sugar, added sugars in HSR algorithm
Other milks	2	2	2	0	2	0	
Cream products							
Mascarpone	9	9	0	0	9	0	
Desserts (crème caramel)	2	2	0	2	2	0	
**Edible oils and oil emulsions**							
*Guideline 3: Limit intake of foods containing saturated fats, added salt, added sugars and alcohol.* *Replace high fat foods which contain predominately saturated fats such as butter, cream, cooking margarine, coconut and palm oil with foods which contain predominately polyunsaturated and monounsaturated fats such as oils, spreads, nut butters/pastes and avocado.*							
Coconut oils	43	43	0	0	43	0	Review ABS table. ADG text suggest avoid.
Cooking oils (e.g., rice bran)	16	16	0	0	16	0	Review ABS table
Edible oils (e.g., margarine)	11	11	0	0	11	0	Review ABS table
Cooking spray oils (e.g., coconut)	3	3	0	0	3	0	
**Eggs**	-	-	-	-	-	-	
*Guideline 2: Choose lean meat and poultry, fish, eggs and/or plant-based alternatives*							
**Fish and fish products**							
*Guideline 2: Choose lean meat and poultry, fish, eggs and/or plant-based alternatives* *Fresh, frozen and canned varieties of meats, poultry or fish are all suitable, but choose varieties that are low in salt and saturated fat. Processed meats such as salami, mettwurst, bacon and ham are not part of this food group. They are classified as discretionary choices because they are high in saturated fat and/or salt.*							
Chilled fish							
Smoked salmon	82	1	82	0	82	0	Review ABS table
Other chilled raw fish	7	3	4	1	6	1	
Canned fish							
Anchovies	24	2	24	0	24	0	Review ABS table
Canned herring	9	0	8	0	8	0	
Canned salmon	5	0	3	0	3	2	
Other canned fish	8	5	4	0	8	0	
Other fish products not specified	28	9	24	0	27	1	Review ABS table
**Fruit and vegetables**							
*Guideline 2: Tuck into vegetables and fruit. Fresh, frozen, canned or dried varieties of vegetables and fruit are all suitable foods. Check the ingredients list and choose varieties of canned vegetables without added salt and canned fruit in natural juice, not syrup* *Vegetables and fruit to limit: …dried fruit can also stick to the teeth and increase the risk of tooth decay. For this reason…dried fruit should be consumed only occasionally and in small amounts.* *The intake of some salted, dried, fermented or pickled vegetables has been associated with increased risk of some cancers, so intake of these foods should be limited. Also limit intake of fried vegetables such as potato and vegetable chips and crisps, which add extra kilojoules and salt. Chips and crisps are included in ‘discretionary choices’*							
Fruit							
Dried fruit	54	50	3	43	54	0	Review ABS table and ADG text on dried fruit
Fruit-based products (e.g., date balls)	20	5	1	18	20	0	Review ABS table and ADG text on dried fruit
Coconut chunks	10	10	4	4	10	0	Review ABS table (coconut products)
Fruit in syrup	3	3	0	3	3	0	
Vegetables							
Sundried tomatoes	6	1	6	4	6	0	
Other veg. products (namkeem, fried shallot)	3	2	1	0	3	0	
Nuts and seeds							
Nuts, salted and sweet-coated	6	6	3	3	6	0	
Herbs and spices							
Herb pastes	32	0	32	14	32	0	Review ABS table. Potential new category
Spice mixes	43	9	38	5	43	0	Review ABS table. HSR impacted by as prepared.
Curry powders	4	0	4	0	4	0	
**Meat and Meat Products**							
*Guideline 2: Choose lean meat and poultry, fish, eggs and/or plant-based alternatives* *Fresh, frozen and canned varieties of meats, poultry or fish are all suitable, but choose varieties that are low in salt and saturated fat. Processed meats such as salami, mettwurst, bacon and ham are not part of this food group. They are classified as discretionary choices because they are high in saturated fat and/or salt.*							
Pate and meat spreads	51	49	17	0	51	0	Review ABS table
Raw flavoured meats	41	38	20	2	41	0	Review ABS table. Further meat categories needed
Sausages and hot dogs	37	0	37	1	37	0	Review ABS table. Consider salt cut-off
Meat not otherwise specified	15	10	12	0	15	0	Review ABS table. Further meat categories needed
Raw flavoured meats	8	3	6	0	8	0	
Uncoated frozen/chilled processed meat	6	2	5	0	6	0	
Kebabs	3	2	2	0	3	0	
Burgers	3	0	3	0	3	0	
**Non-alcoholic beverages**							
*Guideline 2: Tea and coffee provide water, although they are not suitable for young children and large quantities can have unwanted stimulant effects in some people.* *Guideline 3: Limit intake of foods containing saturated fats, added salt, added sugars and alcohol.*							
Teas (not plain)	42	2	0	9	10	32	Review ABS table. HSR failures suggest review added sugars
Fruit juices	4	0	0	3	3	1	
**Sauces, dressings, dips and spreads**							
Guideline 3: Limit intake of foods high in saturated fats, added salt, added sugars and alcohol.							
Vinegars	11	0	4	8	11	0	Review ABS table
**Special foods**							
*Not specifically covered*							
Meal replacements	75	21	46	70	75	0	Review ABS table and eligibility for HSR. HSR impacted by ‘as prepared’
**Sugars, honey and syrups**							
*Guideline 3: Limit intake of foods high in saturated fats, added salt, added sugars and alcohol.* *Foods and drinks that are artificially sweetened can provide a useful alternative to those high in added sugars.*							
Sweeteners	20	0	0	20	20	0	Review ABS table and ADG text on artificial sweeteners
**TOTAL**	**2219**	**1136**	**1159**	**538**	**2116**	**103**	

* For the purposes of this Appendix we have extracted direct statements from the Australian Dietary Guidelines Summary Document: https://www.nhmrc.gov.au/_files_nhmrc/file/your_health/healthy/nutrition/n55a_australian_dietary_guidelines_summary_131014_1.pdf.

(b) Discretionary Outliers with HSR ≥ 3.5

**Major Food Categories *(Extracts of Relevant ADG text *)***	****n****	**Saturated Fat Traffic Light Red**	**Sodium Traffic Light Red**	**Total Sugar Traffic Light Red**	***n*** **ADG Failure**	***n*** **HSR Failure**	**Policy Recommendation (Where *n* ≥ 10)**
**Breads and bakery**							
*Guideline 2: Go for wholegrains. Wholemeal or wholegrain varieties are preferable because they provide more dietary fibre, vitamins and minerals than refined grain (cereal) foods...* *Grain (cereal) foods which have high amounts of added saturated fats, added sugars, and/or salt such as most cakes, muffins, pies, pastries and biscuits are not included in this group but are classified ‘discretionary’ choices.*							
Bread							
Savoury breads	36	1	2	0	33	3	Mostly healthier options of discretionary foods
Sweet breads	17	0	1	4	12	5	Mostly healthier options of discretionary foods
Others (garlic bread, ice cream cone, taco shell)	25	0	0	0	25	0	None. Healthier options of discretionary foods
Biscuits							
Savoury biscuits (crackers and crispbreads)	20	0	2	1	17	3	Mostly healthier options of discretionary foods Review ABS table and kJ cut-off savoury biscuits
Sweet unfilled biscuits (plain, fruit and nut)	17	0	0	5	12	5	Mostly healthier versions of discretionary foods
Plain dry biscuits	12	0	0	0	12	0	None. Healthier options of discretionary foods
Cakes, Muffins and Pastries							
Pastries (filo sheets, quiches)	24	6	1	0	17	7	Mostly healthier versions of discretionary foods
Cake mixes	8	0	1	0	7	1	
Cakes	8	0	0	1	7	1	
**Cereal and Grain Products**							
*Guideline 2: Go for wholegrains. Wholemeal or wholegrain varieties are preferable because they provide more dietary fibre, vitamins and minerals than refined grain (cereal) foods...* *Grain (cereal) foods which have high amounts of added saturated fats, added sugars, and/or salt such as most cakes, muffins, pies, pastries and biscuits are not included in this group but are classified ‘discretionary’ choices.*							
Cereal and nut-based bars							
Cereal bars	158	1	1	22	134	24	Mostly healthier versions of discretionary foods HSR failures suggest review sugar, added sugars
Nut-based bars	46	3	0	12	32	14	Mostly healthier versions of discretionary foods HSR failures suggest review sugar, added sugars
Puff-based bars	9	0	0	1	8	1	
Ready-to-eat breakfast cereals							
Sweet, cocoa-based and puff cereals	38	0	0	25	13	25	HSR failures suggest review sugar, added sugars Review ABS table. Sugar cut-off for breakfast cereals (30/100 g, 35/100 g with fruit) already high.
Breakfast cookie/rusk	9	0	0	1	8	1	
Other cereal products							
Stuffing mixes	2	0	0	0	2	0	Healthier versions of discretionary foods
**Confectionery**							
*Guideline 3: Limit intake of foods high in saturated fats, added salt, added sugars and alcohol.* *Limit intake of foods and drinks containing added sugars such as confectionary (sic)*							
Chewing gums	75	0	0	0	75	0	Review application of HSR
Jelly	63	0	0	7	57	7	Mostly healthier versions of discretionary foods HSR failures suggest review sugar, added sugars
Chocolate and sweets	36	4	0	1	31	5	Mostly healthier versions of discretionary foods HSR failures suggest review saturated fat
**Convenience foods**							
*Guideline 2: Eat a wide variety of nutritious foods from the five food groups…* *Guideline 3: Limit intake of foods containing saturated fats, added salt, added sugars and alcohol.*							
Ready meals							
Frozen ready meals	180	22	24	7	136	44	Many healthier versions of discretionary foods Review ABS table: complexity of classification highlights diversity of products. HSR failures suggest review salt and saturated fat
Ambient ready meals	127	10	22	4	98	29	As above
Chilled ready meals	97	50	21	6	40	57	As above
Others (meal kits)	8	2	3	1	4	4	
Soup							
Dry Soup mixes (as prepared with water)	96	0	0	2	94	2	ABS table review HSR impacted by ‘as prepared’
**Dairy**							
*Guideline 2: Include milk, yoghurt and cheese and/or alternatives—mostly reduced fat* *Some other milk products, such as ice-cream, can be relatively high in saturated fat and added sugars, so are classified under discretionary choices, together with cream and butter.*							
Ice creams and edible ices							
Edible ices	28	0	0	2	26	2	None. Healthier options of discretionary foods
Ice creams	16	0	0	1	15	1	None. Healthier options of discretionary foods
Frozen yoghurt	4	0	0	2	2	2	
Soy-based ice cream	2	0	0	0	2	0	
Desserts							
Rice puddings	31	0	0	27	4	27	HSR failures suggest review sugar, added sugars
Other prepared desserts, mousses	18	0	0	0	18	0	None. Healthier options of discretionary foods
Dessert mixes	9	1	0	1	7	2	
Milks							
Probiotic drinks	9	0	0	5	4	5	
**Edible oils and oil emulsions**							
*Guideline 3: Limit intake of foods containing saturated fats, added salt, added sugars and alcohol.* *Replace high fat foods which contain predominately saturated fats such as butter, cream, cooking margarine, coconut and palm oil with foods which contain predominately polyunsaturated and monounsaturated fats such as oils, spreads, nut butters/pastes and avocado.*							
Edible oils	2	1	0	1	0	2	
**Eggs**	-	-	-	-	-	-	
**Fish and fish products**							
*Guideline 2: Choose lean meat and poultry, fish, eggs and/or plant-based alternatives* *Fresh, frozen and canned varieties of meats, poultry or fish are all suitable, but choose varieties that are low in salt and saturated fat. Processed meats such as salami, mettwurst, bacon and ham are not part of this food group. They are classified as discretionary choices because they are high in saturated fat and/or salt.*							
Processed fish							
Frozen fish	219	0	13	0	131	13	Review ABS table Most are healthier versions of discretionary foodsHSR failures suggest review salt
**Fruit and vegetables**							
*Guideline 2: Tuck into vegetables and fruit. Fresh, frozen, canned or dried varieties of vegetables and fruit are all suitable foods. Check the ingredients list and choose varieties of canned vegetables without added salt and canned fruit in natural juice, not syrup.* *Vegetables and fruit to limit: …dried fruit can also stick to the teeth and increase the risk of tooth decay. For this reason…dried fruit should be consumed only occasionally and in small amounts.* *The intake of some salted, dried, fermented or pickled vegetables has been associated with increased risk of some cancers, so intake of these foods should be limited. Also limit intake of fried vegetables such as potato and vegetable chips and crisps, which add extra kilojoules and salt. Chips and crisps are included in ‘discretionary choices’.*							
Vegetables							
Pickled vegetables	155	1	35	3	117	38	Many healthier options of discretionary foods HSR failures suggest review salt
Frozen potato products	125	1	0	0	124	1	Review ABS table ‘Potato Products’ Healthier options of discretionary foods
Fruit							
Fruit bars and bites	52	4	0	46	5	47	HSR failures suggest review sugar, added sugars
Jams and marmalades	4	0	0	0	4	0	
Seasonings	11	0	0	0	11	0	Review ABS table
**Meat and meat products**							
*Guideline 2: Choose lean meat and poultry, fish, eggs and/or plant-based alternatives.* *Fresh, frozen and canned varieties of meats, poultry or fish are all suitable, but choose varieties that are low in salt and saturated fat. Processed meats such as salami, mettwurst, bacon and ham are not part of this food group. They are classified as discretionary choices because they are high in saturated fat and/or salt.*							
Frozen and chilled meats							
Coated/breaded/frozen meats	152	8	17	0	128	24	Mostly healthier options of discretionary foods HSR failures suggest review salt, saturated fat
Meat with pastry	57	36	6	0	20	37	Some healthier options of discretionary foods HSR failures suggest review saturated fat, salt
Sliced meats	104	0	84	0	20	84	Some healthier options of discretionary foods HSR failures suggest review salt
Canned meats	46	1	11	0	34	12	Many healthier options of discretionary foods HSR failures suggest review salt
Salami and cured meats	8	2	1	0	5	3	
Burgers	7	7	0	0	0	7	
Dried meats	6	0	0	0	6	0	
Bacon	4	0	3	0	1	3	
**Non-alcoholic beverages**							
*Water is essential for life. Choose water instead of drinks with added sugars or alcohol…Consumption of drinks with added sugars, such as soft drinks and cordials, fruit drinks, vitamin waters, energy and sports drinks can increase risk of excessive weight gain in both children and adults. Water has an advantage over these drinks, and also over fruit juice and artificially sweetened soft drinks…*							
Hot chocolate, milk flavourings	22	0	0	15	15	7	HSR impacted by as prepared HSR failures suggest review sugar, added sugars
Beverage mixes	6	0	0	1	5	1	
Electrolyte drinks	2	0	0	0	2	0	
**Sauces, dressings, spreads and dips**							
*Guideline 3: Limit intake of foods containing saturated fats, added salt, added sugars and alcohol.* *Tips to eat less saturated fat: … cut down on dishes with cream, buttery or creamy sauces or fatty gravy, instead choose tomato-based dishes.* *Limit foods high in added sugars including… sweetened sauces and dressings…*							
Sauces							
Pasta sauces							
Tomato-based pasta sauces	389	2	15	1	371	18	Mostly healthier options of discretionary foods Review ABS table, tomato sauces home vs commercial HSR failures suggest review salt
Cream-based pasta sauces	37	4	4	0	29	8	Many healthier options of discretionary foods HSR failures suggest review saturated fat, salt
Meat-based pasta sauces	15	0	1	0	14	1	None. Mostly healthier options of discretionary foods
Meal-based sauces							
Ambient meal-based sauces	84	4	1	5	75	9	Mostly healthier options of discretionary foods HSR impacted by as prepared
Powdered meal-based sauces	44	4	7	1	33	11	Many healthier options of discretionary foods HSR impacted by as prepared
Liquid recipe bases	41	1	14	0	26	15	Many healthier options of discretionary foods HSR impacted by as prepared HSR failures suggest review salt
Curry pastes	11	0	1	1	9	2	Mostly healthier options of discretionary foods
Gravies and stocks	16	0	0	0	16	0	Mostly healthier options of discretionary foods HSR impacted by as prepared
Table sauces	54	0	11	15	28	26	Some healthier options of discretionary foods HSR failures suggest review salt, sugar, added sugars
Meat accompaniments	24	0	1	3	20	4	Mostly healthier options of discretionary foods HSR failures suggest review sugar, added sugars
Spreads and dips							
Dips							
Vegetable- based chilled dips (hummus, tzatziki, guacamole)	292	9	22	1	262	30	Mostly healthier options of discretionary foods HSR failures suggest review salt, saturated fat
Salsa	68	0	16	0	52	16	Mostly healthier options of discretionary foods HSR failures suggest review salt
Savoury spreads							
Relishes, pickles and chutneys	124	0	19	57	48	76	Some healthier options of discretionary foods HSR failures suggest review salt, sugar, added sugars
Other savoury spreads	7	0	2	0	5	2	
Other spreads	15	3	0	8	4	11	HSR failures suggest review sugar, saturated fat
Salad dressings and vinegars	10	0	0	0	10	0	None. Healthier options of discretionary foods
**Snack foods**							
*Guideline 2: Vegetables and fruit to limit…Limit intake of fried vegetables such as potato and vegetable chips and crisps, which add extra kilojoules and salt. Chips and crisps are included in ‘discretionary choices’* *Guideline 3: Limit intake of foods containing saturated fats, added salt, added sugars and alcohol.*							
Potato chips	246	2	46	0	198	49	Many healthier options of discretionary foods HSR failures suggest review salt, FVNL points
Other snackfoods	127	21	30	5	80	47	Many healthier options of discretionary foods HSR failures suggest review salt, sat fat, FVNL points
Corn chips	91	17	17	0	57	34	Many healthier options of discretionary foods HSR failures suggest review salt, sat fat, FVNL points
Snack packs	23	0	0	0	23	0	Review ABS table definitions
Vege-based snacks	62	5	32	5	24	38	Some healthier options of discretionary foods HSR failures suggest review salt, saturated fat, sugar, FVNL points
Popcorn	55	2	2	1	50	5	Mostly healthier options of discretionary foods
**Special foods**							
*Not specifically covered in ADG text.*							
Special foods	2	0	0	0	2	0	
**Sugars, honey and related products**							
*Guideline 3: Limit intake of foods high in saturated fats, added salt, added sugars and alcohol.* *Limit foods high in added sugars including…syrups…*							
Syrups	14	0	0	5	5	9	HSR failures suggest review sugar
Sugars	1	0	0	0	0	1	
**TOTAL**	**4105**	**235**	**510**	**296**	**3130**	**975**	

* For the purposes of this Appendix we have extracted direct statements from the Australian Dietary Guidelines Summary Document: https://www.nhmrc.gov.au/_files_nhmrc/file/your_health/healthy/nutrition/n55a_australian_dietary_guidelines_summary_131014_1.pdf.
